# Associations between Melatonin, Neuroinflammation, and Brain Alterations in Depression

**DOI:** 10.3390/ijms23010305

**Published:** 2021-12-28

**Authors:** Eunsoo Won, Kyoung-Sae Na, Yong-Ku Kim

**Affiliations:** 1 Department of Psychiatry, Chaum, Seoul 06062, Korea; eunsooowon@gmail.com; 2 Department of Psychiatry, CHA Bundang Medical Center, CHA University, Seongnam 13496, Korea; 3 Department of Psychiatry, Gachon University Gil Medical Center, Incheon 21565, Korea; ksna13@gmail.com; 4 Department of Psychiatry, Korea University Ansan Hospital, Korea University College of Medicine, Ansan 15355, Korea

**Keywords:** melatonin, neuroinflammation, major depressive disorder, biomarker

## Abstract

Pro-inflammatory systemic conditions that can cause neuroinflammation and subsequent alterations in brain regions involved in emotional regulation have been suggested as an underlying mechanism for the pathophysiology of major depressive disorder (MDD). A prominent feature of MDD is disruption of circadian rhythms, of which melatonin is considered a key moderator, and alterations in the melatonin system have been implicated in MDD. Melatonin is involved in immune system regulation and has been shown to possess anti-inflammatory properties in inflammatory conditions, through both immunological and non-immunological actions. Melatonin has been suggested as a highly cytoprotective and neuroprotective substance and shown to stimulate all stages of neuroplasticity in animal models. The ability of melatonin to suppress inflammatory responses through immunological and non-immunological actions, thus influencing neuroinflammation and neurotoxicity, along with subsequent alterations in brain regions that are implicated in depression, can be demonstrated by the antidepressant-like effects of melatonin. Further studies that investigate the associations between melatonin, immune markers, and alterations in the brain structure and function in patients with depression could identify potential MDD biomarkers.

## 1. Introduction

Recently, changes in the immune system that lead to neuroinflammation and subsequent alterations in brain regions involved in emotional regulation have been suggested to have a central role in the pathophysiology of major depressive disorder (MDD) [1]. In previous studies, immune system dysfunction has been reported, demonstrated by pro-inflammatory conditions in patients with MDD [2,3,4,5]. The association between increased inflammatory markers and depression can be explained by the neurotoxic effects of neuroinflammation on specific brain regions involved in emotion regulation [6]. In addition, previous imaging studies of patients with depression have reported changes in the structure or function of brain areas in pro-inflammatory states [7].

A prominent feature of MDD is a disruption of sleep–wake cycles. In addition, research has identified a complex bidirectional link between sleep and depression, and a common finding in depressed patients is a habit of sleeping at a time that is out of phase with the body’s biological rhythms [8]. Extensive literature has shown that sleep disruption is an underlying component of the pathophysiology of depression [9], with a majority of patients complaining of insomnia or hypersomnia [10,11]. Sleep has been divided into rapid eye movement (REM) and non-rapid eye movement (NREM) sleep based on polysomnography signals, with a minimum of 20% high-voltage, low-frequency cortical δ waves present in NREM sleep. Therefore, NREM sleep is also referred to as slow-wave sleep [12]. Studies that have used sleep electroencephalograms have shown characteristic changes in NREM and REM sleep in depression, such as sleep continuity disruption, decreases in non-rapid eye movement sleep production and rapid eye movement (REM) sleep latency, and increases in REM sleep duration and frequency [11,13].

The central pacemaker of the circadian rhythm is the suprachiasmatic nucleus (SCN) of the hypothalamus [14], which synchronizes physiological and behavioral rhythms with 24 h periodicity of the light–dark cycle, with light being the primary stimulus for orienting the SCN-induced rhythms with the external environment [12]. The relationship between depressive-like behavior and disruption of biological rhythms has been widely reported in both animal and human studies [15]. Rodents exposed to short photoperiods showed depression-like behavior [16] and lower dopaminergic and somatostatin neuron levels in the hypothalamus [17]. Disruption of central SCN rhythms was associated with helplessness, despair, and anxiety-like behaviors [18]. In clinical studies, moderate changes in the timing of the sleep–wake cycle were shown to have profound effects on subsequent mood [19] and the rhythmicity of specific mood-related symptoms and behaviors was associated with a risk for psychiatric disorders [20].

The SCN generates a neural output signal that induces melatonin synthesis from the pineal gland in dimmed light, and melatonin is considered one of the most important moderators of circadian rhythm [21]. The sleep–wake cycle is the most overt circadian rhythm and is highly affected by melatonin as a physiological sleep regulator [12]. Concerning the pathophysiology of depression, dysfunction in monoamine neurotransmission has been most widely studied, with current antidepressant treatment concentrated on normalization of monoamines, such as serotonin, norepinephrine, and dopamine [22]. Because serotonin is the precursor of melatonin, and norepinephrine and dopamine influence melatonin production [23], given the central role of melatonin in sleep, several clinical studies have investigated the association between melatonin level and depression. However, results have been inconsistent and reported lower serum or saliva levels of melatonin, no difference in levels, or even higher levels in depressed individuals compared to controls [24,25,26,27,28,29]. Although the association between melatonin and depression has been explained more widely by regulatory effects of melatonin on sleep and the circadian rhythm, the inconsistencies of previous studies suggest that melatonin could potentially influence mood through diverse physiological actions, in addition to regulating the circadian rhythm.

Melatonin has been reported to influence various immunomodulatory actions in both in vivo and in vitro models [30]. Melatonin has also been shown to have cytoprotective properties [31] that are related to its immunoregulatory effects and its antioxidant and scavenging capacities [32]. Melatonin can inhibit pro-oxidant enzyme synthesis, facilitate antioxidant enzyme synthesis, and protect from oxidative damage [33]. Melatonin has also been suggested to exert cytoprotection through membrane stabilization [34]. The γ-aminobutyric acid (GABA)-ergic system might be associated with melatonin’s neuroprotective actions, as one study has suggested that melatonin extends protection to neurons by activating GABAergic receptors [35]. Melatonin’s anti-excitotoxic activity has also been reported in previous studies [36]. Melatonin has been reported to reduce neuronal damage in animal models of Alzheimer’s disease (AD) [37] and Parkinson’s disease (PD) [38], along with brain trauma [39,40], focal ischemia [41], cadmium [42,43], δ-aminolevulinic acid [44] toxicity, hyperbaric hyperoxia [45,46], γ radiation [47], and several neurotoxins [48].

In this review, we evaluated the influence of melatonin on the immune system and neuroinflammation, along with the cytoprotective properties, which might influence brain structure and function, contributing to the pathophysiology of MDD.

## 2. The Immune System and Neuroinflammation in Depression

The hypothalamus secretes corticotropin-releasing hormone in response to stress, and these neurons project from the paraventricular nucleus of the hypothalamus to the noradrenergic centers of the brainstem and spinal cord [49]. The locus coeruleus of the brainstem sends direct projections to the sympathetic and parasympathetic preganglionic neurons in the spinal cord [49], increases sympathetic activity, and decreases parasympathetic activity through α1- and α2-adrenoceptors, respectively [50,51]. The principal neurotransmitters of the autonomic nervous system (ANS) are norepinephrine (NE) and acetylcholine (ACh) [52], and the sympathetic nervous system (SNS) primarily acts by releasing NE, whereas the parasympathetic nervous system (PNS) mainly uses ACh as its neurotransmitter [53,54]. The SNS controls epinephrine (E) and NE biosynthesis and secretion from the adrenal medulla [55]. In response to stress, the SNS is activated and stimulates medullary cells to release E and NE into the blood [56]. When the stressor is terminated, the PNS is activated and ACh is released to stimulate the muscarinic receptors of the target organs [57]. However, when a stressful situation persists, as in MDD, the SNS continues to be activated without normal counteraction of the PNS and the catecholamine level can increase while the acetylcholine level decreases [58]. Catecholamines modulate cytokine release through α- and β-adrenoceptors on immune cells [59]. NE has been shown to enhance the production of TNF-α [60,61], and E and NE have both been shown to stimulate the production of IL-6 [62]. NE has also been shown to augment macrophage phagocytosis and tumoricidal activity [63]. In comparison, ACh has been shown to inhibit the production of TNF-α along with other pro-inflammatory cytokines, such as IL-1β, IL-6, and IL-18 [64], and is considered to be immunoinhibitory [65]. Therefore, in chronically stressful situations, such as MDD, continuous sympathetic activity together with insufficient parasympathetic activity can increase catecholamine levels and decrease acetylcholine levels to increase pro-inflammatory cytokine levels. Accordingly, depression-like symptoms can be induced by pro-inflammatory cytokines and reversed by pro-inflammatory cytokine receptor antagonists [66]. The association between immune activation and depression has been repeatedly suggested [67,68,69], along with improvement in depressive symptoms with pro-inflammatory cytokine antagonist treatment [70]. Studies on medically healthy subjects have reported an association between depressed mood and increased pro-inflammatory cytokine production, and studies on patients with MDD have reported increased levels of pro-inflammatory cytokines, such as IL-1β, IL-6, and TNF-α [2,3,4]. Furthermore, c-reactive proteins (CRPs) are increased following IL-6 secretion [5] and have been reported to be elevated in patients with MDD [71,72,73]. The link between increased inflammatory markers and depression can be explained by the toxic effects of neuroinflammation on specific brain regions involved in depression.

Increases in pro-inflammatory cytokines can influence the brain by increasing neurotoxic metabolites through the kynurenine pathway or by directly exerting neurotoxic effects on specific brain regions [7]. Pro-inflammatory cytokines activate the kynurenine pathway, resulting in an increase in neurotoxic kynurenine pathway metabolites, including 3-hydroxykynurenine, 3-hydroxyanthranilic acid, and quinolinic acid [74], and decreases in the neuroprotective kynurenine pathway metabolite kynurenic acid [75]. The potent free radical donor, 3-hydroxykynurenine, promotes oxidative stress, which can lead to neuronal apoptosis [76,77]. In addition, 3-hydroxyanthranilic acid produces reactive hydrogen peroxide and hydroxyl radicals by auto-oxidation [78]. Quinolinic acid triggers NMDA receptors, stimulates glutamate release, inhibits glutamate reuptake, and reduces glutamine synthetase action, which lead to increased extracellular glutamate and excitatory neuron activation, which cause excitotoxicity and apoptosis [79,80]. Quinolinic acid also induces mitochondrial dysfunction, cytochrome c release, ATP exhaustion, free radical formation, and oxidative damage [81]. Furthermore, brain areas important for emotional regulation can be directly influenced by excess activation of brain cytokine networks; further, pro-inflammatory cytokines can decrease neurotrophic support and neurogenesis through brain-derived neurotrophic factor signaling pathway downregulation [82,83,84], reduce cell proliferation through the nuclear factor-kappa B signaling pathway [85], and increase the glutamate level to result in excitotoxicity and reduced neurogenesis through NMDA receptor activation [86,87].

A previous study investigated the association between serum concentrations of kynurenine pathway metabolites and hippocampal and amygdala volumes in patients with MDD; the kynurenic acid/quinolinic acid ratio was positively correlated with hippocampal and amygdala volumes [88]. Another study evaluated the impact of changes in the glucocorticoid and inflammatory systems and how they affect hippocampal volumes in MDD, reporting a negative effect of IL-6 concentration on the hippocampal volume [89]. Another study investigated neuroinflammation in individuals with late-life depression and reported elevated CRP levels and hippocampal structural reductions [90]. In a study that investigated whether increased inflammation in major depression affects corticostriatal reward circuitry, increased CRP levels were associated with decreased connectivity between the ventral striatum and ventromedial prefrontal cortex (vmPFC) and striatum and vmPFC connectivity was associated with increased plasma IL-6, IL-1β, and IL-1 receptor antagonists [91].

Therefore, conditions that influence systemic inflammation and subsequent neuroinflammation and those that can induce alterations in brain regions related to emotional regulation might contribute to the pathophysiology of MDD. As melatonin has immunomodulatory and cytoprotective actions, disruption of the melatonin system has been implicated in MDD.

## 3. Melatonin Synthesis and Secretion

Melatonin is a neurohormone primarily produced by the pineal gland [92] and also by other organs, including the cerebellum, skin [93], retina, Harderian gland [94], lymphocytes [95], platelets [96], bone marrow cells [97], and the gastrointestinal (GI) tract [98]. Particularly in the GI tract, enterochromaffin cells synthesize and secrete melatonin into circulation based on food (tryptophan) intake and melatonin concentrations in the GI tract are several hundred times higher than that in the blood or the pineal gland [98,99]. GI melatonin can be released into circulation, especially under conditions of high dietary tryptophan levels [31,98]. The melatonin level remains relatively stable until 35–40 years of age and then gradually decreases, with minimal difference in day and night levels at ≥65 years of age [100,101,102].

The first step in melatonin formation is the uptake of L-tryptophan from circulation into the pineal gland [100]. L-tryptophan is hydroxylated by tryptophan-5-hydroxylase into 5-hydroxytryptophan and then decarboxylated by 5-hydroxytryptophan decarboxylase into serotonin. Serotonin is acetylated by arylalkylamine-*N*-acetyltransferase into *N*-acetylserotonin and then methylated by acetylserotonin-*O*-methyltransferase into melatonin; arylalkylamine-*N*-acetyltransferase is the rate-limiting enzyme for this process [99,103,104]. Once synthesized, melatonin is released into the bloodstream instead of being stored in pineal cells [105]. Because norepinephrine increases adenylate cyclase activity, noradrenergic projections to the pineal gland control arylalkylamine-*N*-acetyltransferase activity, which subsequently enhances arylalkylamine-*N*-acetyltransferase activity [23]. Circulating melatonin is metabolized primarily in the liver and secondarily in the kidney. Melatonin is hydroxylated to 6-hydroxymelatonin, followed by sulfate conjugation to 6-hydroxymelatonin sulfate (90%) or glucuronide conjugation to 6-hydroxymelatonin glucuronide (10%), with approximately 5% of serum melatonin being excreted unmetabolized through urine. Minor metabolites of melatonin include cyclic 2-hydroxymelatonin, *N*-gamma-acetyl-*N*-2-formyl-5-methoxykynurenamine, and *N*-gamma-acetyl-5-methoxykynurenamine [31,100].

The synthesis and secretion of melatonin are influenced by light, with darkness increasing and light inhibiting both processes. Melatonin is the main circadian output marker of the brain, along with cortisol, and its synthesis and secretion are regulated by light through retinal ganglion cells and melanopsin. Photosensory information arrives at the pineal gland through a polyneuronal pathway that includes the retinohypothalamic tract, which projects from the retina to the SCN, the major circadian oscillator [31,92,100,106]. Fibers from the SCN influence the intermediolateral horn cells of the spinal cord, where preganglionic sympathetic neurons that innervate the superior cervical ganglion are located. Subsequently, the postganglionic sympathetic fibers terminate on the pinealocytes and regulate melatonin synthesis by releasing norepinephrine [31,106].

Melatonin is released predominantly at night because light suppresses the activity of enzymes that synthesize it [107]. Light passes through the retina, and information is transferred to the SCN of the hypothalamus and eventually to the pineal gland [108]. Rhythmic synthesis and secretion of melatonin are generated by the circadian pacemaker situated in the SCN and synchronized based on the 24 h light–dark cycle [100]. Melatonin secretion begins with the dimming of light (sundown), with the onset usually around 9–10 pm., gradually increases and reaches its peak around 2–3 am., and gradually decreases until sunrise [109]. During the night, approximately 80% of the melatonin is synthesized and maintained in the serum at a concentration of 80–120 pg/mL, compared with a daytime serum concentration of 10–20 pg/mL [99,100]. The half-life of serum melatonin is less than 60 min [31,101,110]. Endogenous oscillators within the SCN control the circadian production of pineal melatonin based on the environmental light–dark cycle [111].

Pineal melatonin is released into the third ventricle and then into general circulation [92]. Melatonin has several targets, mainly G-protein-coupled receptors that are classified into three groups: MT1 (Mel1a), MT2 (Mel1b), and GPR50 (mammals)/Mel1c (non-mammals) [112,113,114,115]. Melatonin has high affinity for MT1 and MT2 but not for GPR50 in mammals; as an agonist of these receptors, it leads to G protein activation and beta-arrestin recruitment [112,116,117]. In humans, MT1 is located on chromosome 4q35.1 and MT2 is located on chromosome 11q21-22, with 60% homology to MT1 [100,113,118]. MT1 inhibits adenylate cyclase and stimulates inositol phosphate, and MT2 inhibits cyclic adenosine monophosphate (cAMP) and cyclic guanosine monophosphate (cGMP) formation [100,119,120]. The vitamin D receptor, a nuclear receptor, has been reported to bind melatonin directly [121], as have enzymes such as quinone reductase 2, metalloprotease-9, pepsin, and protein phosphatase 2 [122,123,124,125]. In mammals, high-affinity melatonin receptors are primarily located in the hypothalamus and pars tuberalis of the anterior lobe of the pituitary gland in the brain [100]. MT1 is distributed in the retina, the cornea, the anterior pituitary, the SCN, the cortex, the amygdala, the hippocampus, the thalamus, substantia nigra, nucleus accumbens, and the cerebellum [99,126]. MT2 is distributed in the retina, the cortex, the hippocampus, the paraventricular nucleus, and the cerebellum [99,127]. Melatonin receptors are also present in peripheral tissues, including the heart, lungs, the liver, the gallbladder, the adrenal gland, the small intestine, kidneys, the prostate, breasts, ovaries, the uterus, adipocytes, the skin, T lymphocytes, and B lymphocytes [99,107].

## 4. Biological Effects of Melatonin

Melatonin functions as an endogenous synchronizer, and melatonin synthesis is rapidly suppressed even in the dark phase by acute light exposure of sufficient intensity [100,128]. In numerous studies, melatonin has been shown to synchronize circadian rhythms when binding to MT-receptors in the SCN [107]. Melatonin delayed circadian rhythms when it was administered in the morning and advanced circadian rhythms when administered in the afternoon or early evening [129]. Melatonin was also shown to readjust after acute light–dark phase shifts, such as jet lag and shift work [130]. Furthermore, administration of melatonin was shown to entrain circadian rhythms in most blind individuals that had free-running rhythms [131]. Inhibition of dopamine release by melatonin has been demonstrated in specific areas of the mammalian central nervous system, including the hypothalamus, the hippocampus, the medulla-pons, and the retina, and the interaction of melatonin with the dopaminergic system has been hypothesized to have a significant role in nonphotic and photic entrainment of the biological clock [132].

Melatonin secretion has also been suggested to be important in regulating memory processes [133]. The presence of melatonin receptors in the human hippocampus has been widely acknowledged [134], with MT1 predominantly present in the cornus ammonis (CA)1 subfield [135] and MT2 in the CA4 and CA3 subfields [136]. Melatonin has been shown to have both enhancing and inhibitory effects on the excitability of hippocampal neurons [133,136,137], along with vasoactive effects in the hippocampus [138]. Other biological effects of melatonin include antiepileptic effects [139,140,141,142,143] via antioxidant activities [144], increase in γ-aminobutyric acid (GABA) concentration [145] and GABA receptor affinity [146], and decreases in *N*-methyl-d-aspartate (NMDA) excitatory levels [147]. However, proconvulsant effects of melatonin have also been reported [148].

## 5. Melatonin and Inflammation

A bidirectional communication system has been identified, with melatonin acting on the immune system, and vice versa [149]. As the pineal gland is an immune target, cytokines such as interferon-gamma (IFN-γ) [150] and tumor necrosis factor-alpha (TNF-α) [151] have been shown to influence the melatonin level. Numerous studies have suggested endogenous synthesis of melatonin by the immune system [152], considered a source of extra-pineal melatonin [153,154]. Furthermore, melatonin receptors have been shown to be present in the immune cells of various species [155,156]. In comparison, melatonin has been shown to modulate immune responses [157,158]. In both normal and immunosuppressed conditions, melatonin has been shown to exert immune-enhancing effects, such as increasing the number of immune-related cells (including macrophages [159]; NK cells [160]; interleukin (IL)-1β [161], IL-6, and IL-12 [162]; splenic CD4(+) cells [163]; IgG1 and IgM [164]; and IL-2 and IFN-γ [165]). However, in conditions with exacerbated immune responses, melatonin decreased immune reactions, represented by a reduction in neutrophil infiltration [166], migration of neutrophils and monocytes [167], and levels of cyclooxygenase-2 (COX-2) and nitric oxide synthase (iNOS) [168]. Additionally, numerous animal and clinical studies have indicated that melatonin mitigates pro-inflammatory cytokine production [169,170,171,172,173,174].

Immunomodulatory actions of melatonin have been widely reported [30], and melatonin has been found to have either pro- or anti-inflammatory effects [149,175], depending on the systemic condition. In normal conditions and immunosuppressed conditions, melatonin was shown to exert immune-enhancing effects. A study that examined the response of macrophages/microglia to multiple injections of melatonin in the pineal gland and different regions of the brain reported a significant increase in macrophage/microglia cellularity [159]. Further, melatonin injection was shown to induce natural killer (NK) cell activity [160] and humans treated with melatonin showed an increased neutrophil chemotactic response, with melatonin suggested as having a relevant role during the tissue leukocyte infiltration in inflammatory and immune responses [176]. Melatonin administration to normal and immune-depressed mice significantly increased antibody responses and restored impaired T-helper cell activity [177]. Melatonin was also suggested to exert an additive effect on the modulation of phagocytic function [178]. Melatonin stimulated the production of IL-2 and IL-1β, which stimulated cell immunity [161], and melatonin administration after trauma-hemorrhage was shown to significantly improve depressed immune functions, which was confirmed by the restoration of peritoneal macrophage IL-1 and IL-6 release, as well as significantly improve splenocyte IL-2 and IL-3 release and splenocyte proliferative capacity [179]. Melatonin activated human Th1 lymphocytes by increasing the production of IL-2 and IFN-γ in vitro [162].

In comparison, in exacerbated immune response states, melatonin has predominantly shown immune-dampening effects, with various studies reporting a reduction in pro-inflammatory cytokine levels in models of high- or medium-grade inflammation [180,181,182,183,184]. Melatonin attenuated TNF-α, IL-1β, and IL-6; promoted plasma levels of anti-inflammatory cytokine IL-10; and reduced polymorphonuclear neutrophil infiltration in heatstroke rats [166]. The beneficial effect of melatonin on acute pancreatitis was related to a decline in pro-inflammatory cytokines such as TNF-α and stimulation of anti-inflammatory IL-10, along with a decrease in neutrophil infiltration [185]. In rats with transient focal cerebral ischemia, intravenous administration of melatonin decreased the emigration of circulatory neutrophils and macrophages/monocytes into the injured brain and inhibited focal microglial activation [167]. Another study suggested that the reduction in vascular permeability induced by local melatonin injection mediated a reduction in the ability of endothelial cells to interact with neutrophils [186]. Melatonin has also been shown to inhibit lipopolysaccharide (LPS)-induced cyclooxygenase-2 (COX2) and inducible nitric oxide synthase (INOS) protein levels [168] and was suggested to have anti-inflammatory functions through down-regulation of chemokine expression by inhibiting nuclear factor kappa-light-chain-enhancer of activated B cells (NF-κB) in an LPS-stimulated BV2 murine microglial cell line [187]. Melatonin also suppressed IL-8 production in human pulmonary fibroblasts [188] and inhibited LPS-mediated production of TNF-α and IL-8 in neutrophils [189]. Additionally, numerous animal and clinical studies have shown that melatonin mitigates pro-inflammatory cytokine production in inflammatory states, including TNF-α, IFN-γ, IL-1β, IL-2, IL-6, IL-8, and IL-12 [166,169,170,171,172,173,174,190,191,192,193,194,195,196,197,198,199,200,201,202,203,204,205,206,207,208,209,210] (Table 1). However, in some studies, melatonin did not prevent increases in pro-inflammatory cytokines in models of high-grade inflammation [211,212].

Melatonin has also been shown to suppress inflammatory responses through non-immunological actions, such as antioxidative protection and mitochondrial function preservation, which promote antioxidative processes as well as decrease free radical formation and excessive NO formation [213,214,215,216,217,218,219,220,221,222,223,224,225,226]. Melatonin was reported to inhibit inflammatory cell activation by reducing myeloperoxidase activity [214,227] and suppressing the inflammasome nucleotide-binding oligomerization domain, a leucine-rich family, and the pyrin domain-containing-3 (NLRP3) [228,229,230,231]. Although a general association between melatonin and anti-inflammatory effects might be an oversimplification [232], overall, melatonin can have pro-inflammatory properties in immune suppression or basal conditions and anti-inflammatory properties in pro-inflammatory conditions [233,234,235]. Therefore, disruption of the melatonin system can further contribute to the exacerbation of inflammation in pro-inflammatory conditions, such as in MDD.

## 6. Melatonin and the Brain

Melatonin has been suggested to be a highly neuroprotective substance that can exert cytoprotective effects [236,237] through biological mechanisms that are separate from its immunomodulatory actions, such as regulating oxidative stress, apoptosis, and mitochondrial homeostasis [238]. Melatonin is a potent antioxidant [239] and has been shown to decrease the extracellular level of glutamate [240] to maintain Ca_2_^+^ homeostasis and prevent Ca_2_^+^-dependent cell injury [241]. One study confirmed that melatonin reduced free radical generation by acting on the MT2 [242] and decreased oxidative stress damage by reducing Nox2 and Nox4 expression [243]. In an animal model of Alzheimer’s disease (AD) and patients with AD, melatonin was reported to act as a potent antioxidant [37], reduced Aβ-mediated oxidative stress and lipid peroxidase [244,245], regulated levels of mRNA that encoded antioxidant enzymes [192], inhibited nicotinamide adenine dinucleotide phosphate oxidase formation, and reduced reactive oxygen species production [246]. In animal models of Parkinson’s disease (PD), melatonin was shown to downregulate oxidative stress effects, act as a potent antioxidant [38], interfere with lipid peroxidation in the hippocampus and striatum, inhibit neuronal death in the nigrostriatal area [247], elevate antioxidant enzyme levels in the nigrostriatal pathway [248], counteract mitochondrial oxidative phosphorylation enzyme reduction in the substantia nigra [249], and reduce nigrostriatal dopaminergic degeneration and lipid peroxidation [250].

In animal studies, melatonin has stimulated all stages of neuroplasticity [251], including neurogenesis, synaptogenesis [252,253,254], axogenesis [255], and dendritogenesis [256]. Melatonin was shown to repair hippocampal dendrite loss by increasing Ca_2_^+^/calmodulin levels and activation of Ca_2_^+^/calmodulin (CaM)-dependent kinase II [256]. MT2 receptor activation, which induces Akt/GSK-3β/CRMP-2 signaling, mediates functional axonogenesis and synaptic formation in central neurons [255]. In an animal model of sporadic AD, melatonin significantly increased hippocampal synaptic density and the number of excitatory synapses, decreased the number of inhibitory synapses, upregulated pre- and post-synaptic proteins, improved the ultrastructure of neuronal and glial cells, and reduced glial density [257]. Melatonin was also shown to attenuate synaptic dysfunction and reduce astrogliosis [258]. In animal models of ischemia, melatonin promoted the proliferation of endogenous oligodendrocyte progenitor cells, alleviated white matter (WM) damage [259], promoted subsequent myelination in WM [260], and significantly improved WM lesions and gliosis [261]. Melatonin also promoted distal dendritic ramifications in the layer II/III cortical pyramidal cells of rats exposed to toluene vapors [262] and promoted the genesis of CA1 cells in rats with pinealectomy-induced hippocampal cell loss [263].

Disruption of the melatonin system can lead to a decrease in its cytoprotective and neuroprotective effects, which can further contribute to the neurotoxic effects on brain regions involved in emotion regulation and that contribute to symptoms of depression.

## 7. Melatonin and Depression

Melatonin has been strongly implicated in the pathophysiology of MDD. In a study that investigated the effects of genetic deletion of the MT1 and/or MT2 receptors on depression- and anxiety-like behaviors in C_3_H/HeN mice, MT1 and/or MT2 receptor deletion reportedly caused a deficit in hedonic and social interaction behavior and increased anxiety-like behavior; the authors suggested that MT1 and/or MT2 melatonin receptor dysregulations were involved in depression and anxiety pathophysiology [264]. One study reported a possible association between the melatonin plasma level and a neuroinflammatory state in depression [265] and a genetic variation in *N*-acetylserotonin *O*-methyltransferase, which is a key enzyme in melatonin biosynthesis [266]. A single nucleotide polymorphism, rs713224, located near the brain-expressed melatonin receptor (MTNR1A) gene, was associated with somatic complaints of depression symptoms on the Center for Epidemiological Studies Depression (CES-D) scale [267], indicating that patients with MDD have weaker responses to melatonin [251].

Agomelatine, an MT1 and MT2 agonist and a 5-HT2C and 5-HT2B serotonin receptor antagonist, is used to treat MDD [268] and has been shown to have positive phase-shifting properties, such as inducing sleep phase advancement, body temperature decline, and melatonin onset. The antidepressant effects of agomelatine prompted preclinical research to validate the antidepressant effects of melatonin [251] using numerous studies with animal models of depression, such as the forced swimming test, the tail suspension test, and the chronic mild stress test, and showed evidence for antidepressant-like actions of melatonin [269,270,271,272,273]. These antidepressant-like effects of melatonin have been associated with neurotransmitter systems, such as the serotoninergic, glutamatergic, and GABAergic systems, along with hypothalamic–pituitary–adrenal axis modulation [269,271,272]. The antidepressant effects of melatonin remain unclear in human studies, although several studies have reported that melatonin use is beneficial for improving symptoms associated with depression [274,275].

A possible mechanism of melatonin’s anti-depressant effects is its ability to modulate neuroplastic responses in the hippocampus. The hippocampus is the most widely studied brain area related to depression, with a wide range of meta-analytic evidence indicating structural and functional abnormalities of the hippocampus in depression [276], and inflammatory processes have been shown to further contribute to structural changes in the hippocampus in depression [277,278]. Preclinical evidence has shown that the hippocampus is one of the main targets for melatonin actions in the brain [251], with melatonin promoting distal dendritic ramifications in layer II/III cortical pyramidal cells [262] and preventing hippocampal CA1 and CA3 cell loss [263]. Melatonin has also been shown to stimulate neurogenesis, axogenesis, and dendritogenesis of hippocampal neurons [254,255,279]. Agomelatine, which mainly acts on the melatonin receptor, has been reported to modulate hippocampal plasticity in animal models of depression [280,281,282,283], similar to other antidepressants. The disruption of the melatonin system that further contributes to the exacerbation of inflammation and decreases in cytoprotective and neuroprotective effects of hippocampal cells might be one of the core mechanisms underlying the pathophysiology of depression.

## 8. Conclusions

**Figure 1:**
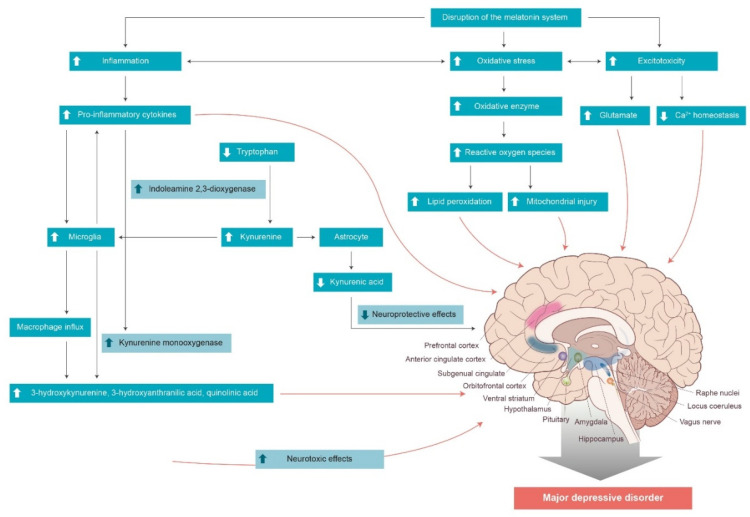
Disruption of the melatonin system can exacerbate inflammatory conditions, which increase neurotoxic metabolites through the kynurenine pathway or directly exert neurotoxic effects on specific brain regions. Disruption of the melatonin system can also lead to oxidative stress and excitotoxicity, further contributing to neuroinflammation, as well as exerting direct neurotoxic effects. Neuroinflammation and subsequent alterations in brain regions involved in emotional regulation have been suggested as an underlying mechanism for the pathophysiology of major depressive disorder.

The ability of melatonin to suppress inflammatory responses through immunological and non-immunological actions, thus influencing neuro-inflammation and subsequent alterations in brain regions implicated in depression (Figure 1), is supported by its antidepressant-like effects. Furthermore, disturbances in the circadian system and mood symptoms have been widely suggested to precede by years, the emergence of characteristic cognitive and motor symptoms of neurodegenerative diseases, including AD, PD, and Huntington’s disease, and contribute to the onset of the disease [284,285,286,287,288]. The therapeutic potential of melatonin has been investigated in neurodegenerative diseases, although the effects on sleep quality and activity rhythms have been inconsistent [289,290,291,292,293], possibly due to methodological inconsistencies across trials [294]. Future studies that assess the associations between melatonin, immune markers, and alterations of brain structure and function in patients with depression will aid us to not only better understand potential biomarkers of MDD but also gain insight into ways for the early diagnosis and prevention of neurodegenerative diseases. Longitudinal clinical studies that investigate the effects of melatonin on the long-term progression of cognitive and motor symptoms of neurodegenerative diseases and mood symptoms of depressive disorders can provide clues on novel types of pharmacological interventions. A wide variety of ani-inflammatory agents have been investigated for their possible anti-depressant effects, including celecoxib as a cyclooxygenase-2-selective nonsteroidal anti-inflammatory drug [295,296,297], TNF-α inhibitors [70,298,299], aspirin (acetylsalicylic acid) [300], and minocycline (tetracycline antibiotic) [301]. Further studies on the treatment efficacy of melatonin along with other therapeutic agents that enhance melatonin’s anti-inflammatory, anti-oxidant, and cytoprotective properties can help to develop methods to detect and ultimately treat depression.

## Figures and Tables

**Table 1:** Melatonin and pro-inflammatory cytokine production in an inflammatory state.

Inflammatory State	Effect of Melatonin Administration	Species	Reference
Heatstroke-associated multiple organ dysfunction syndrome resembling septic shock	Attenuates TNF-α, IL-1β, and IL-6	Rodent	[166]
Overexpression of inflammatory mediators induced in the heart by acute exercise	Prevents increase in TNF-α, IL-1, and IL-6 mRNA	Rodent	[169]
Cerulein-induced acute pancreatitis	Reduces the expression of TNF-α, IL-1β, IL-6, and IL-8	Rodent	[170]
Aerosolized pancreatic fluid introduced into airways to induce inflammation	Reduces mRNA and protein expression of TNF-α	Rodent	[171]
Intracerebroventricular administration of LPS	Attenuates TNF-α and IL-1β	Rodent	[172]
Duchenne muscular dystrophy	Attenuates IL-1β IL-2, IL-6, TNF-α, and IFN-γ	Human	[173]
LPS administration to pregnant mice	Attenuates the LPS-evoked elevation of TNF-α in maternal serum and fetal brain	Rodent	[174]
Respiratory distress syndrome	Attenuates TNF-α, IL-6, and IL-8	Human	[190]
Endotracheal intubation	Attenuates IL-6, IL-8, and IL-12	Human	[191]
Alzheimer’s transgenic mice	Attenuates TNF-α in the hippocampus	Rodent	[192]
Generation of chronic gastric ulcers by indomethacin	Blocks increase in the expression of TNF-α, IL-1β, and IL-8	Rodent	[193]
Radiation-induced lung injury	Reduces the elevation of TNF-α expression	Rodent	[194]
Bacillus Calmette–Guérin/LPS-induced hepatic injury	Attenuates increase in TNF-α and IL-1β	Rodent	[195]
Mechlorethamine-induced nephrotoxicity	Attenuates increase in TNF-α and IL-1β	Rodent	[196]
Hypoxia-induced retinal ganglion cell death	Reverses the upregulation of TNF-α and IL-1β	Rodent	[197]
Acute lung ischemia-reperfusion injury	Attenuates TNF-α	Rodent	[198]
Escherichia-coli-induced pyelonephritis	Attenuates increase in TNF-α	Rodent	[199]
Taurocholate-induced acute pancreatitis	Reduces TNF-α	Rodent	[200]
Colitis induced by intracolonic instillation of dinitrobenzene sulfonic acid	Reduces the expression of TNF-α	Rodent	[201]
Periodontitis	Reduces TNF-α and IL-1β	Rodent	[202]
Colitis established by intrarectal injection with 2,4,6-trinitrobenzenesulfonic acid and ethanol	Reduces TNF-α and IL-1β	Rodent	[203]
Dimethylnitrosamine-induced liver injury	Decreases the expression of TNF-α, IL-1β, and IL-6	Rodent	[204]
Hemorrhagic shock	Suppresses the release of TNF-α and IL-6	Rodent	[205]
Acetic-acid-induced colitis	Attenuates increases in TNF-α, IL-1β, and IL-6	Rodent	[206]
FK506-induced renal oxidative stress	Reduces TNF-α and IL-6	Rodent	[207]
Streptozotocin-induced diabetic neuropathy	Reduces elevated levels of TNF-α and IL-6	Rodent	[208]
Brain-contusion-induced oxidative insult	Reduces upregulation of IL-6	Rodent	[209]
Zucker diabetic fatty rats	Lowers TNF-α, IL-6, and CRP	Rodent	[210]
Hepatic ischemia-reperfusion injury	Promotes TNF-α and IL-6 release	Rodent	[211]
LPS treatment	Has no effect on TNF-α or IL-1β release	Rodent	[212]
